# Histomorphological and Immunophenotypic Features of Pill-Induced Esophagitis

**DOI:** 10.1371/journal.pone.0128110

**Published:** 2015-06-05

**Authors:** Ji Won Kim, Byeong Gwan Kim, Su Hwan Kim, Won Kim, Kook Lae Lee, Sun-ju Byeon, Euno Choi, Mee Soo Chang

**Affiliations:** 1 Department of Internal Medicine, Seoul National University Boramae Hospital, Seoul National University College of Medicine, Seoul, Republic of Korea; 2 Department of Pathology, Seoul National University Boramae Hospital, Seoul National University College of Medicine, Seoul, Republic of Korea; University of Palermo, ITALY

## Abstract

The aim of this study was to investigate histomorphological and immunophenotypic features in pill-induced esophagitis. We comparatively evaluated the histomorphological, immunophenotypic features of pill-induced esophagitis vs. reflux esophagitis, as well as clinical information and endoscopic findings. Fifty-two tissue pieces from 22 cases of pill-induced esophagitis, 46 pieces from 20 reflux esophagitis, and 16 pieces from 14 control samples were subjected to immunohistochemistry for inflammatory infiltrates (CD3 for T lymphocyte, CD20 for B lymphocyte, CD56 for NK cell, CD68 for macrophage, CD117 for mast cell) and eosinophil chemotaxis-associated proteins (Erk, leptin, leptin receptor, pSTAT3, phospho-mTOR). As a result, Histomorphology showed that a diffuse pattern of dilated intercellular spaces was more frequently observed in pill-induced esophagitis, while reactive atypia and subepithelial papillary elongation were more often found in reflux esophagitis (*P* < 0.05, respectively). Interestingly, intraepithelial eosinophilic microabscess, intraepithelial pustule and diffuse pattern of dilated intercellular spaces were observed in 14% (3 cases), 9% (2 cases) and 32% (7 cases) of pill-induced esophagitis, respectively, but in no cases of reflux esophagitis. Regarding intraepithelial inflammatory infiltrates in pill-induced esophagitis, T lymphocytes were the most common cells, followed by eosinophil; 11 and 7 in one x400 power field, respectively. Intraepithelial pSTAT3-positive pattern was more frequently observed in pill-induced esophagitis than in reflux esophagitis, at 45% (10 cases) versus 10% (2 cases), respectively (*P* < 0.05). Considering the distal esophageal lesion only, intraepithelial pustule, diffuse dilated intercellular spaces and stromal macrophages were more frequently found in distal pill-induced esophagitis, whereas reactive atypia and intraepithelial mast cells in reflux esophagitis (*P* < 0.05, respectively). In conclusion, diffuse dilated intercellular spaces, intraepithelial eosinophil microabscess, pustule, T lymphocytes, eosinophils, and pSTAT3 positivity can be added to histopathological features of pill-induced esophagitis, other than non-specific ulcer. Besides, distal pill-induced esophagitis may be histopathologically differentiated from reflux esophagitis.

## Introduction

Pill-induced esophagitis first reported by Pemberton in 1970, described a patient who felt stuck in her chest and experienced retrosternal pain after taking a potassium chloride tablet; esophageal ulcer was finally confirmed in the patient on endoscopy [1]. Since then, more than 1,000 cases of pill-induced esophagitis have been reported [2,3]. The authors described more than 100 different causative drugs, endoscopic findings and tissue pathology based on hematoxylin and eosin (H&E)-staining features. The incidence of pill esophagitis is unknown, but the best estimate is 3.9/100,000/year from a Swedish survey of 700,000 patients for 4 years at medical institutions[4,5]. There are few original articles on clinical and endoscopic features of pill-induced esophagitis [6,7]. Some authors summarized the case reports in medical literature [5] or reviewed features of pill-induced esophagitis [3,8]. Additionally, some review articles described the medication-associated damage on gastrointestinal tract, including the esophagus [2,9–13]. However, histopathological features of pill-induced esophagitis have only been described as nonspecific ulcer [13] or intraepithelial eosinophilic infiltration [14]. Moreover, to the best of our knowledge, there was no study of immunophenotypic features.

Histologically, intraepithelial eosinophilic infiltration is one of the intriguing features of pill-induced esophagitis [14]. Reflux esophagitis and eosinophilic esophagitis are the more well-recognized disease entities involving intraepithelial eosinophilic infiltration [14–17]. Additionally, esophageal ulceration is commonly observed in pill-induced and reflux esophagitis. Pill-induced esophagitis and reflux esophagitis account for more than 1/5^th^ and 2/3^rd^ esophageal ulceration cases, respectively [18]. Recent reports suggest that intraepithelial eosinophil infiltration within the esophagus is induced throughout multiple signaling pathways, including extracellular signal-related kinase (ERK) [19–21], leptin [22] and leptin signaling-related proteins, such as the signal transducer and activator of transcription 3 (STAT3) and the mammalian target of rapamycin (mTOR) [23–26].

In the present study, we evaluated histomorphological and immunophenotypic features of pill-induced esophagitis that have hitherto received little attention, and compared the findings between pill-induced and reflux esophagitis.

## Materials and Methods

### Case collection

This is a retrospective study, which included 22 patients of pill-induced esophagitis with endoscopic biopsy of esophageal mucosal tissue due to ulceration, between 2002 and 2008. The number of study cases was determined based on the following; Histological findings on esophageal tissue of pill-induced esophagitis has been described briefly in the form of case reports, although endoscopic and clinical analysis of pill-induced esophagitis has been studied about many patients, up to 98 patients in a single study [6]. According to Higuchi et al, esophageal ulcers were detected in 88 patients among the biopsied cases of 7,564 at a single hospital for 11 years, and pill-induced esophagitis was diagnosed in 20 patients out of 88 esophageal ulcer-patients [18]. Hence, 20 patients or so would be a sufficient number of cases in order to analyze endoscopic biopsied-tissues of pill-induced esophagitis.

Pill-induced esophagitis was defined as sudden onset of esophageal symptoms after taking a pill, or acute manifestation of esophageal symptoms within < 2 weeks of pill-taking. In fact, the onset time of esophageal symptoms after taking pills has not absolutely defined in pill-induced esophagitis. Abid et al have studied the patients who developed acute esophageal symptoms within 3 days after taking pills [6], and Boyce described that the onset time of esophageal symptoms was within hours to 10 days after taking pills [8]. In my hospital, endoscopic examination was performed at less than 2 weeks after taking pills and having acute esophageal symptoms.

Additionally, we comparatively evaluated 20 patients of reflux esophagitis. The control group was selected from 14 people of unremarkable esophageal mucosal histology, which were biopsied for the surveillance of small submucosal esophageal tumors. In my hospital, endoscopic biopsy on submucosal tumor is recommended. For instance, carcinoid and granular cell tumor etc. usually involve the mucosal layer, and the diagnosis can be made with endoscopic biopsy. However, some of leiomyoma and gastrointestinal stromal tumor are situated deep in submucosa or proper muscle layer, accordingly, the diagnosis cannot be done with forceps biopsy commonly including only mucosal tissue. This mucosal tissue shows normal histologic features, thus it is used as control tissue.

We reviewed the patient medical records, endoscopic features, and histopathologic features of esophageal mucosal tissue.

### Ethics Statement

All human tissue specimens were obtained during diagnostic and therapeutic process. The participants did not provide the written consent to participate in the present study. The retrospective study was performed using the samples over the shelves after the pathologic diagnosis, and all of the samples were anonymized before the study. The retrospective study protocol was reviewed and approved by the Institutional Review Board of the Seoul National University Boramae Hospital under the condition of the anonymization (IRB No. 20131205/26-2013-125/122).

### Histomorphological evaluation and immunohistochemistry

We examined 52 tissue pieces from 22 pill-induced esophagitis patients, 46 tissue pieces from 20 reflux esophagitis patients, and 16 tissue pieces from 14 control samples. We evaluated the following histomorphological features on H&E-staining. The number of intraepithelial eosinophils was counted at the most infiltrated part, and indicated as number per high power field (one x400 field area: 0.24mm^2^, Olympus BX51), and then, divided into 3 grades: grade 1, 1~9 eosinophils; grade 2, 10~29; grade 3, 30~59. The presence or absence of eosinophilic microabscess was assessed (microabscess: a continuous collection of > 4 eosinophils). Additionally, if dilated intercellular spaces were observed, they were divided into 3 grades i.e. mild (focal, occasional or sporadic small size intercellular spaces), moderate (moderately spread and larger size intercellular spaces) and marked (widespread and very large intercellular spaces) [27], and then, categorized into 2 patterns of focal versus diffuse (including ‘moderate’ and ‘marked’). The presence or absence of vacuolization of squamous epithelial cells, intraepithelial pustule, subepithelial papillary elongation (subepithelial papillary length > 1/2 of total epithelial thickness), and reactive atypia was evaluated. ‘Reactive atypia’ was considered when a squamous epithelial cell had a vesicular nucleus and a prominent nucleolus. The thickening of basal layer was rated as normal: < 25% of total epithelial thickness, mild thickening: 25~50%, and marked: >50% [15–17,28].

Immunohistochemical staining was performed on the Discovery XT automated immunostainer (Ventana Medical Systems, Tucson, AZ, USA). Briefly, 3-μm-thick tissue sections were placed on electrostatic charged glass slides and subjected to online deparaffinization and antigen retrieval. The antigen was detected using the Discovery ChromoMap DAB Detection Kit (Ventana Medical Systems). Primary antibodies used were summarized in Table 1). Inflammatory cells were counted in the most positive portion of the epithelium and stroma, separately. Intraepithelial and stromal immunoreactive cells were denoted as number per high power field (one x400 field area: 0.24mm^2^, Olympus BX51). For eosinophil chemotaxis-associated proteins such as ERK, leptin, leptin-receptor, pSTAT3, and phospho-mTOR, immunoreactive cells were evaluated in the epithelium only.

**Table 1:** Antibodies used in immunohistochemistry.

Antibody	Source	Clone	Dilution	Immunoreaction
Inflammatory cells				
CD3	Novocastra, UK	LN10	x 300	T lymphocyte
CD20	Novocastra, UK	L26	x 2,000	B lymphocyte
CD56	Ventana, USA	123C3	Ready to use	Natural killer cell
CD68	Novocastra, UK	514H12	x 200	Macrophage
CD117	Dako, USA	A4502	x 200	Mast cell
Eosinophil chemotaxis-associated proteins				
Erk1/2 (Thr202/Tyr204)	Cell signaling, USA	D13.14.4E	x 100	Epithelial nucleus
Leptin/Ob	Santa Cruz, USA	A20	x 50	Epithelial cytoplasm
Leptin receptor/Ob-R	Santa Cruz, USA	B3	x 25	Epithelial membrane
Phospho-Stat3 (Tyr705)	Cell signaling, USA	D3A7	x 50	Epithelial nucleus
Phospho-mTOR (Ser2448)	Cell signaling, USA	49F9	x 50	Epithelial cytoplasm

### Statistical analysis

Statistical analyses were carried out using IBM SPSS Statistics version 20.0 (IBM Inc., Chicago, IL, USA). The chi-square test or Fisher’s exact test (2-sided) was used, as appropriate. One-way ANOVA was used to analyze continuous variables. *P* values of < 0.05 were considered statistically significant.

## Results

### Histomorphological features

**Fig 1:**
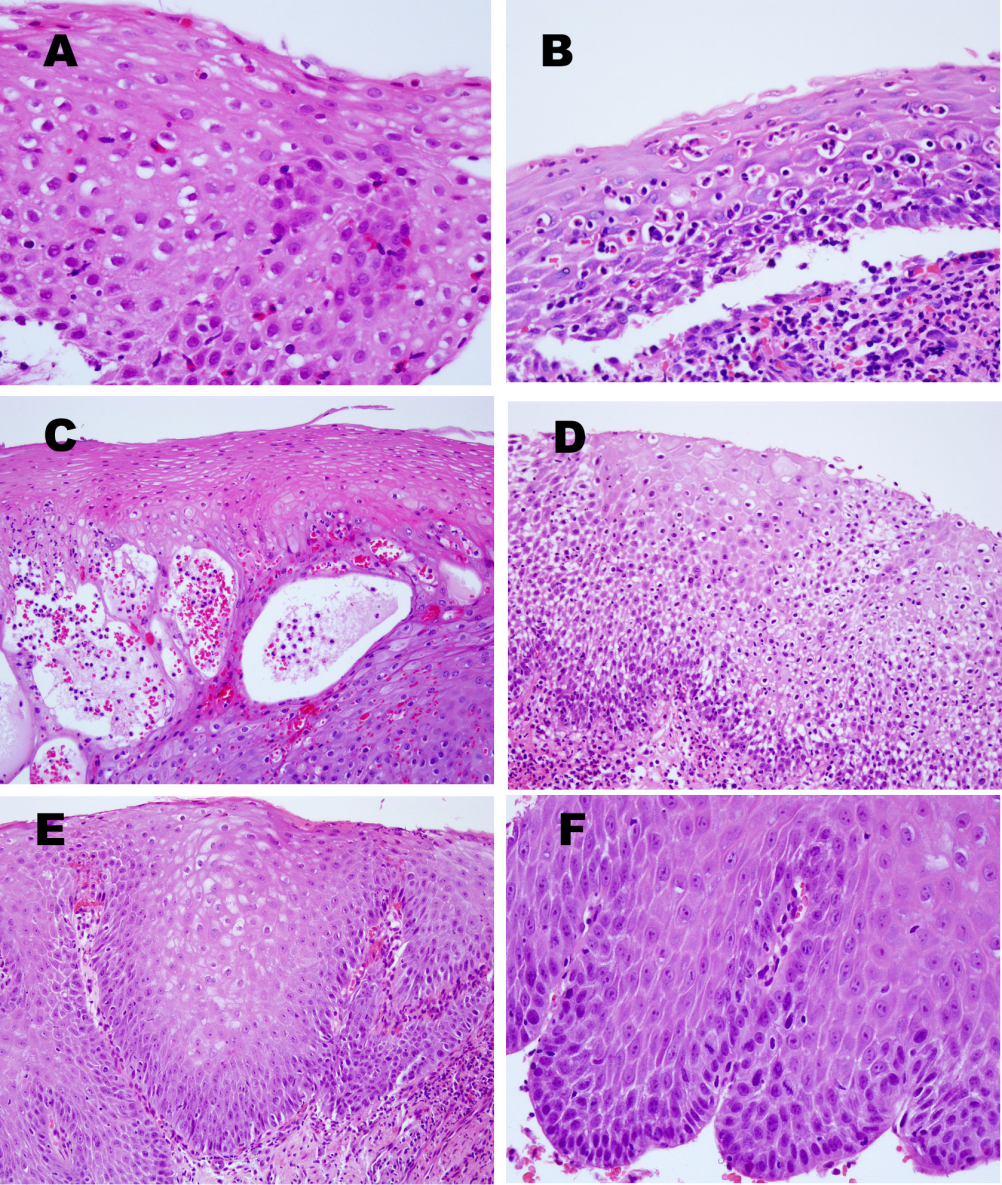
Representative histomorphological features of esophageal mucosal epithelium in pill-induced esophagitis (A-E) and reflux esophagitis (F). (A) This case shows abundant intraepithelial eosinophil infiltration with eosinophilic microabscess with > 4 eosinophils found in rows. (B) Mixed infiltration of eosinophils and neutrophils is observed within the squamous epithelium. (C) Note intraepithelial pustules, i.e., bullae with scattered neutrophils. (D) Esophageal squamous epithelium shows dilated intercellular spaces (upper part), and extensive vacuolization of squamous epithelial cells (lower part). (E) The subepithelial papillae reach upward to approximately three fourths the epithelial thickness. (F) Reactive atypia (vesicular nucleus and prominent nucleolus) of squamous epithelial cells is present. Magnifications: A, B, F, x400 and C, D, E, x200 (H&E stain).

Microscopic features based on H&E were summarized in Table 2, and depicted in Fig 1. Intraepithelial eosinophilic microabscess, intraepithelial pustule and diffuse pattern of dilated intercellular spaces were observed in 14% (3 of 22 cases), 9% (2 cases) and 32% (7 cases) of pill-induced esophagitis, respectively, but in none of reflux esophagitis. Pattern of dilated intercellular spaces was significantly different between the 2 disease groups (*P* = 0.049). Subepithelial papillary elongation was observed in 60% (12/20) of the reflux esophagitis group, but in 23% (5/22) of the pill-induced esophagitis group, respectively (*P* = 0.027). The reactive atypia of squamous epithelial cells (vesicular nucleus and prominent nucleolus) was manifested in 70% (14/20) of the reflux esophagitis group, but in only 1 case of the pill-induced esophagitis group (*P* = 0.000).

**Table 2:** Differentiation of histomorphological features in squamous epithelium between pill-induced esophagitis and reflux esophagitis groups.

	Pill-induced esophagitis		Reflux esophagitis	Control	
	Total (n = 22)	Distal esophagus (n = 5)	Distal esophagus (n = 20)	(n = 14)	^c^ *P* value
Intraepithelial eosinophils, present	16 (73%)	3 (60%)	14 (70%)	4	
^a^grade 1/ grade 2/ grade 3	11/ 4/ 1	3/0/0	10/4/0	4/ 0/ 0	^d^NS, NS
Intraepithelial eosinophil abscess	3 (14%)	1 (20%)	0	0	NS, NS
Intraepithelial neutrophils	5 (50%)	1 (20%)	3 (15%)	0	NS, NS
Intraepithelial pustule	2 (9%)	2 (40%)	0	0	NS, 0.003
Dilated intercellular spaces	12 (55%)	3 (60%)	8 (40%)	1	
focal/ diffuse	5/ 7	1/ 2	8/ 0	1/ 0	0.049, 0.033
Vacuolization of keratinocytes	14 (64%)	2 (40%)	9 (45%)	3	NS, NS
Basal layer hyperplasia	13 (59%)	2 (40%)	11 (55%)	3	
^b^mild/ marked/ basal destruction	3/ 3/ 7	1/0/1	6/ 0/ 5	2/ 0/ 1	NS, NS
Subepithelial papillary elongation	5 (23%)	2 (40%)	12 (60%)	4	0.027, NS
Reactive atypia of squamous cells	1 (5%)	0	14 (70%)	0	0.000, 0.009

Each digit represents the number of cases showing those features in squamous epithelium.

^a^grade 1, 1~ 9 eosinophils; grade 2, 10~29; grade 3, 30~59.

^b^mild, 25~50% of total epithelial thickness; marked, >50%.

^c^The first represents *P* value between total cases of pill-induced esophagitis group and reflux esophagitis group, and the second denotes *P* value between in distal esophagus lesion of both groups.

^d^NS, not significant.

Two histomorphological features of the distal esophagus lesion showed statistical significances between pill-induced and reflux esophagitis. First, a diffuse pattern of intercellular spaces was observed in 40% (2 cases) of pill-induced esophagitis, but in none of all 20 cases of reflux esophagitis (*P* = 0.003). Second, 70% (14 cases) of reflux esophagitis showed reactive atypia, but there were none in the distal pill-induced esophagitis (*P* = 0.009).

### Inflammatory infiltrates, and immunoreactive cells for eosinophil chemotaxis-associated proteins

**Fig 2:**
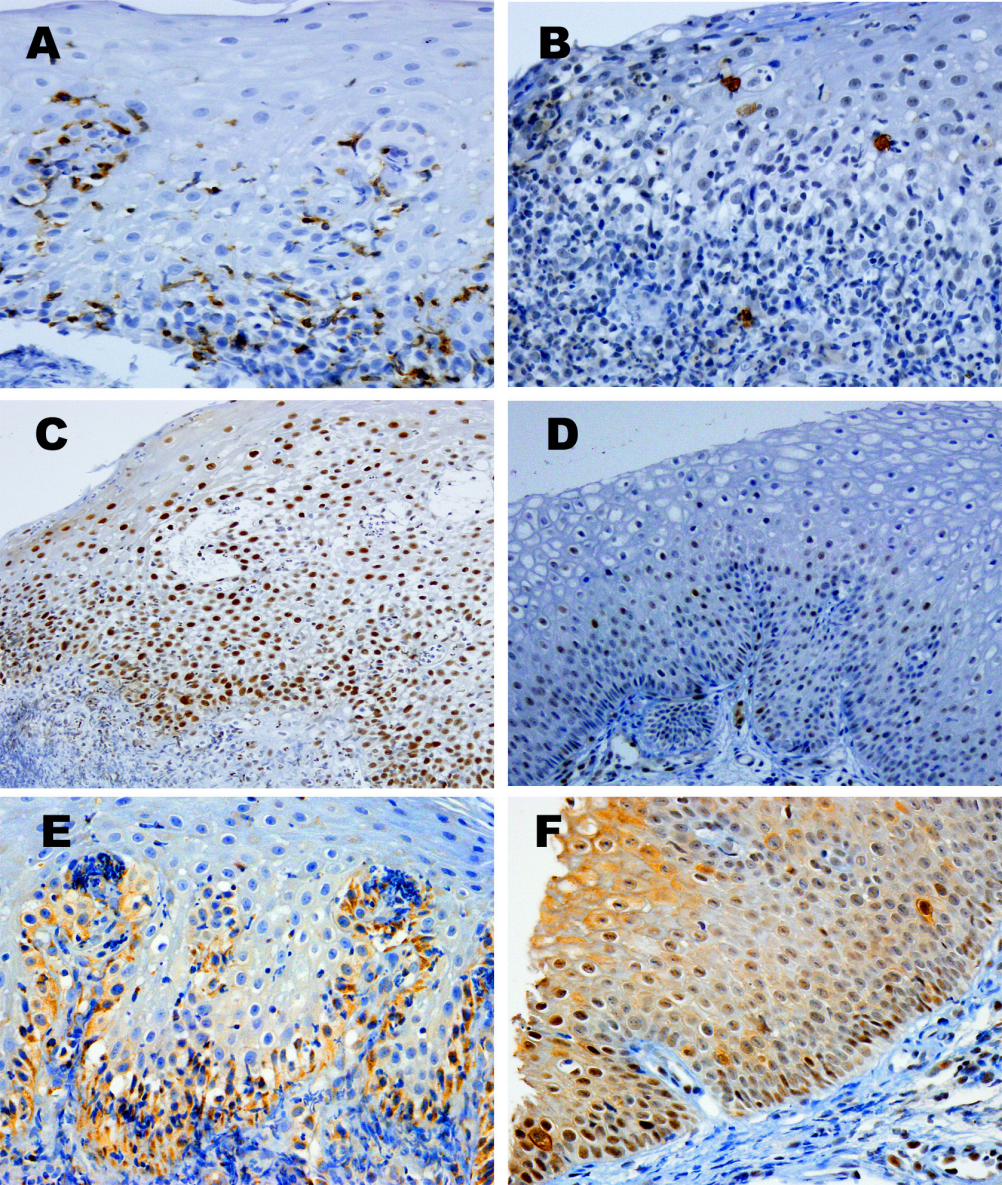
Immunohistochemical microphotographs of esophageal mucosal tissue in pill-induced esophagitis. (A) CD3. T lymphocytes are lodged between squamous epithelial cells. (B) CD117. Intraepithelial mast cells are rarely found. (C) & (D) pSTAT3. Diffuse nuclear staining is exhibited in this case (C), but was negative in another case (D). (E) leptin receptor. This picture shows a negative pattern with normally stained basal cells. (F) phospho-mTOR. Membranous staining is noted in only two cells (at the right middle portion, and the left lower end). Magnifications: A, B, E, F, x400 and C, D x200 (immunohistochemical stain).

Mean number and range of inflammatory cells and immunoreactive cells in each group were listed in Table 3. Representative immunohistochemical features were displayed in Fig 2. T lymphocytes were the most common inflammatory cell among intraepithelial infiltrates, followed by eosinophils.

**Table 3:** Comparison of inflammatory infiltrates and eosinophil chemotaxis-associated cells between pill-induced esophagitis and reflux esophagitis groups.

	Pill-induced esophagitis		Reflux esophagitis	Control	
	Total (n = 22)	Distal esophagus (n = 5)	Distal esophagus (n = 20)	(n = 14)	^c^ *P* value
^a^Intraepithelial, mean number (range)					
Eosinophils	7 (0–56)	1(0–2)	4(0–28)	0 (0–1)	^d^NS, NS
T lymphocytes, CD3 positive	11 (0–30)	8 (0–19)	14 (3–67)	18 (5–55)	NS, NS
B lymphocytes, CD20 positive	2 (0–20)	0 (0–1)	1 (0–4)	0	NS, NS
Mast cells, CD117 positive	2 (0–6)	1 (0–2)	3 (0–7)	2 (0–7)	NS, 0.049
^a^Stromal, mean number (range)					
T lymphocytes, CD3 positive	109 (28–180)	103 (50–130)	97 (15–250)	19 (10–40)	NS, NS
B lymphocytes, CD20 positive	74 (0–290)	87 (0–140)	92 (3–500)	55 (0–150)	NS, NS
NK cells, CD56 positive	1 (0–7)	1 (0–3)	1 (0–3)	1 (0–3)	NS, NS
Macrophages, CD68 positive	14 (0–120)	24 (0–48)	1 (0–4)	0	NS, 0.002
Mast cells, CD117 positive	20 (2–42)	21 (8–32)	24 (0–70)	32 (12–46)	NS, NS
^b^Intraepithelial pSTAT3-positive pattern	10 cases (45%)	2 cases (40%)	2 cases (10%)	0	0.017, NS

^a^Intraepithelial cells and the cells within stroma were displayed as the highest number in one x400 power field (0.24mm^2^ in area).

^b^Intraepithelial pSTAT3-positive pattern was classified into two groups; negative and positive patterns.

^c^The first represents *P* value between total cases of pill-induced esophagitis group and reflux esophagitis group, and the second denotes *P* value between in distal esophagus lesion of both groups.

^d^NS, not significant.

Intraepithelial mast cells were more frequently observed in reflux esophagitis than the pill-induced esophagitis in the distal esophagus, while stromal macrophages were more often found in pill-induced esophagitis (*P* < 0.05). Additionally, intraepithelial eosinophils appeared more in reflux esophagitis than in pill-induced esophagitis without statistical significance.

Intraepithelial pSTAT3 was the only eosinophil chemotaxis-associated protein compared between the 2 disease groups, and pSTAT3-positive pattern was more frequently observed in pill-induced than reflux esophagitis (*P* = 0.017). The pSTAT3 was evaluated in the suprabasal epithelium, because basal cells of the epithelium were stained in all cases, including the control group. The pSTAT3-positive pattern was classified as ‘no staining’, ‘focal staining’, ‘moderate staining’ and ‘marked staining’, and then categorized into 2 tiers: negative (including ‘no staining’ and ‘focal’) versus positive (including ‘moderate’ and ‘marked’).

Among other eosinophil chemotaxis-associated proteins, ERK showed positive staining in the superficial or middle epithelium and/or basal layer of all cases, irrespective of groups. We did not detect any intraepithelial immunoreactivity for leptin. Basal cells alone were stained for leptin-receptor in all cases, including the control group. The phospho-mTOR was considered ‘negative’ i.e., cytoplasmic membranous staining was undetected, or merely observed in a few cells; The latter was found in 10 cases of pill-induced esophagitis, 9 cases of reflux esophagitis and 5 cases of the control group, respectively (S1 Fig).

### Clinical and endoscopic findings

The pill-induced esophagitis group showed an equal gender distribution, whereas reflux esophagitis group showed a male predilection, but it was not statistically significant (Table 4). Pill-induced esophagitis patients (mean: 42.0 years, range: 17–80) were younger than reflux esophagitis patients (mean: 61.2 years, range: 35–91) (*P* = 0.001).The location of lesion was predominantly mid-esophagus with 77% (17 cases) in mid-esophagus and 23% (5 cases) in distal in the pill-induced esophagitis group; whereas all reflux esophagitis were located in the distal esophagus (*P* = 0.000). The esophageal motility test was not performed. There was no clinical condition suspected of esophageal motility disorder in patients, since the patients did not previously suffer from any esophageal symptoms. In the pill-induced esophagitis group, 3 men and 1 woman had single or multiple chronic extra-gastrointestinal diseases, such as hypertension, diabetes mellitus, chronic renal failure, brain ischemia and atrial fibrillation.

**Table 4:** Clinical features of pill-induced esophagitis and reflux esophagitis groups.

	Pill-induced esophagitis	Reflux esophagitis	Control	
	(n = 22)	(n = 20)	(n = 14)	^a^ *P* value
Sex ratio, male: female	11:11	15:05	9:05	^b^NS
Age, mean (range)	42.0 yrs (17–80)	61.2 yrs (35–91)	49.1 yrs (30–73)	0.001
Lesion location, number of cases (%)				0
distal/ mid/ proximal esophagus	5 (23%)/ 17 (77%)/ 0	20 (100%)/ 0/ 0	7 (50%)/ 7 (50%)/ 0	

^a^ *P* value between pill-induced esophagitis and reflux esophagitis groups.

^b^NS, not significant.

Pill-taking history of pill-induced esophagitis group, showed that patients either took 1 or 2 kinds of pills; antibiotics in 11 (50%) patients, non-steroidal anti-inflammatory drug in 9 (41%), antihypertensive drug in 2 (9%), anti-diabetes in 2 (9%), vitamin C in 1 (5%) and digestive [including Stillen (Artemisia asiatica eupatillin), Bioflor (Saccharomyces cerevisiae) Dicetel (pinaverium bromide), and Bearse (digestant)] in 1 (5%). There was no patient taking potassium chloride tablet which is well known to develop a caustic injury. Two (9%) pill-induced esophagitis patients had a history of taking pills just before bedtime. Only three patients had a history of taking pills with no or little water. Most pill-induced esophagitis patients manifested more than a single esophageal symptom i.e. odynophagia in 11 (50%), dysphagia in 9 (41%) and chest pain in 9 patients (41%).

Endoscopically, kissing ulcer (ulcer facing each other) was the most common finding, i.e., in 9 patients (41%) of the pill-induced esophagitis group, and mainly in the mid-esophagus; 8 of the 9 kissing ulcer lesions were located in the mid-esophagus, and the remaining 1, in distal esophagus. Additionally, bleeding was detected in 7 (32%) and esophageal stricture in 2 (9%) patients.

As for treatment, causative drugs were discontinued in all patients. And, two patients were managed with no medication. The rest were treated with a single drug or a couple of drugs; in detail, proton pump inhibitor (omeprazole, esomeprazole, pantoprazole, rabeprazole and lansoprazole) in eleven patients, sucralfate in eight patients, and H2 receptor antagonist (ranitidine and famotidine) in five patients (S1 Table).

The follow-up endoscopy was conducted in 7 (32%) out of 22 patients. The remaining patients rejected it or did not re-visit the hospital, since esophageal symptoms were relieved. The follow-up endoscopy was performed within a couple of months after symptom relief, and they showed unremarkable esophageal mucosa in 5, and half-reduced lesion size in 2 patients.

## Discussion

The present study highlighted immunophenotypes of inflammatory infiltrates in the esophageal mucosal tissue of pill-induced esophagitis with ulceration. To the best of our knowledge, there is no previous report on immunophenotypic study of inflammatory cells, or eosinophil-chemotaxis-associated proteins in pill-induced esophagitis. T lymphocytes (immunoreactive for CD3) were most frequently observed in intraepithelial inflammatory infiltrates in pill-induced esophagitis, followed by eosinophils. T lymphocytes were not easily detected on H& E stain, since they are typically tightly lodged between squamous epithelial cells, however they were detected on immunohistochemical staining. In the distal esophagus lesion, stromal macrophages (immunoreactive for CD68) were more commonly found in pill-induced esophagitis than in reflux esophagitis, while intraepithelial mast cells (immunoreactive for CD117) were more frequent in reflux esophagitis (*P* < 0.05). It is likely that the increase in intraepithelial mast cells is associated with damage from gastric juice mainly hydrochloride, and stromal macrophages with damage from various chemical drugs. Intraepithelial pSTAT3 positive pattern was more frequent in pill-induced esophagitis in the study. Further study is needed to clarify the implications. Meanwhile, other authors reported a positive relationship between eosinophil infiltration and STAT3 in nasal polyp [29] or bronchial epithelial damage by virus infection [30].

The present study particularly emphasized histomorphological features of pill-induced esophagitis, as compared with reflux esophagitis. We observed intraepithelial eosinophil microabscess, intraepithelial pustule and diffuse pattern of dilated intercellular spaces in pill-induced but not reflux esophagitis. These findings can be considered as additional histologic features, although, to date, nonspecific ulcer was the only known histological change in pill-induced esophagitis [13], with pill residue providing the only histologic clue. Interestingly, reactive atypia (vesicular nucleus and prominent nucleolus) in squamous epithelial cells appears to be a unique feature in reflux esophagitis, because it was found in 70% of reflux esophagitis cases, but in only 1 case of pill-induced esophagitis. Additionally, subepithelial papillary elongation was observed more frequently in reflux esophagitis than in pill-induced esophagitis.

We thus identified differential histologic features in the distal esophagus lesion of pill-induced esophagitis and reflux esophagitis. These were intraepithelial pustule, diffuse pattern of ‘dilated intercellular spaces’, and stromal mast cell infiltration for the distal pill-induced esophagitis, whereas reactive atypia of squamous epithelium and intraepithelial mast cell infiltration for reflux esophagitis. In fact, the most commonly recognized site of pill-induced injury to the esophagus is the mid-esophagus, where the aortic arch (at 22 to 24 cm from the incisor teeth) or left atrium (at 30 to 32 cm) impinges upon the esophagus [8]. However, it is important to be aware of histopathological features in the distal esophagus lesion of pill-induced esophagitis. Higushi et al reported that distal pill-induced esophageal ulcer cannot be easily differentiated from the reflux esophagitis ulcer [18]. Furthermore, distal esophageal injury from pills probably occurs more frequently than is clinically recognized because injuries at that site are usually attributed to reflux [5]. De Groen et al reported the location of 20 pill-induced esophagitis, as pan-esophageal lesion in 5 patients, mid or mid-distal in 8, and distal esophageal lesion in 7 [31]. Indeed, experimental studies in normal volunteers have shown that the distal esophagus just above the gastroesophageal junction is the most common site for pills to lodge [32].

The present study disclosed clinical characteristics of pill-induced esophagitis. Most of the current clinical and endoscopic findings were similar to previous reports with a mean patient age of 42 years [33]; odynophagia, dysphagia and chest pain were frequent esophageal symptoms; antibiotics were the most common causative drug and there was a predominance of mid-esophagus lesions [5,6]. The present study showed equal gender distribution in pill-induced esophagitis, while reflux esophagitis revealed male predominance (3 fold higher than females). Other authors reported that pill-induced esophagitis occurred more commonly in female than male patients [6]. Kissing ulcer was the most common endoscopic finding in the present study, but Abid et al reported that the kissing ulcer was observed in 15% of pill-induced esophagitis, i.e., in 7 of 48 patients with ulcer [6]. This difference may arise from patient selection. The present study included only patients with esophageal tissue biopsy, rather than patients diagnosed by clinical and endoscopic findings alone (without tissue biopsy).

In conclusion, eosinophil microabscess, intraepithelial pustule, diffuse pattern of ‘dilated intercellular space’ and pSTAT3 positive pattern might be acceptable histopathological features of pill-induced esophagitis. T cells were the most common inflammatory cells, followed by eosinophils in intraepithelial infiltrates of pill-induced esophagitis., Differential histopathological features observed in distal esophagus lesions included intraepithelial pustule, diffuse dilated intercellular spaces and increased infiltration of stromal macrophages in the distal pill-induced esophagitis, whereas reactive atypia and increased intraepithelial mast cells infiltration in reflux esophagitis.

## Supporting Information

S1 Fig Additional representative pictures of immunohistochemical staining for Erk, leptin, phospho-mTOR, CD20, CD56 and CD117. ERK is stained in the superficial or middle epithelium. There is no intraepithelial positive for leptin. A few intraepithelial cells display cytoplasmic membranous staining for phospho-mTOR. Only one intraepithelial cell shows positive for CD20. As for stromal stain, CD56 and CD117 positive are observed in a few stromal cells.
(TIF)

S1 Table List of treatment medicine in pill-induced esophagitis. (DOC)

S1 File Raw data of pill-induced esophagitis study. (XLSX)
