# Supplementary figures and images for: Histomorphological and Immunophenotypic Features of Pill-Induced Esophagitis
Source: PLoS One. 2015 Jun 5;10(6):e0128110. doi: 10.1371/journal.pone.0128110 (PMC4457729; doi:10.1371/journal.pone.0128110)

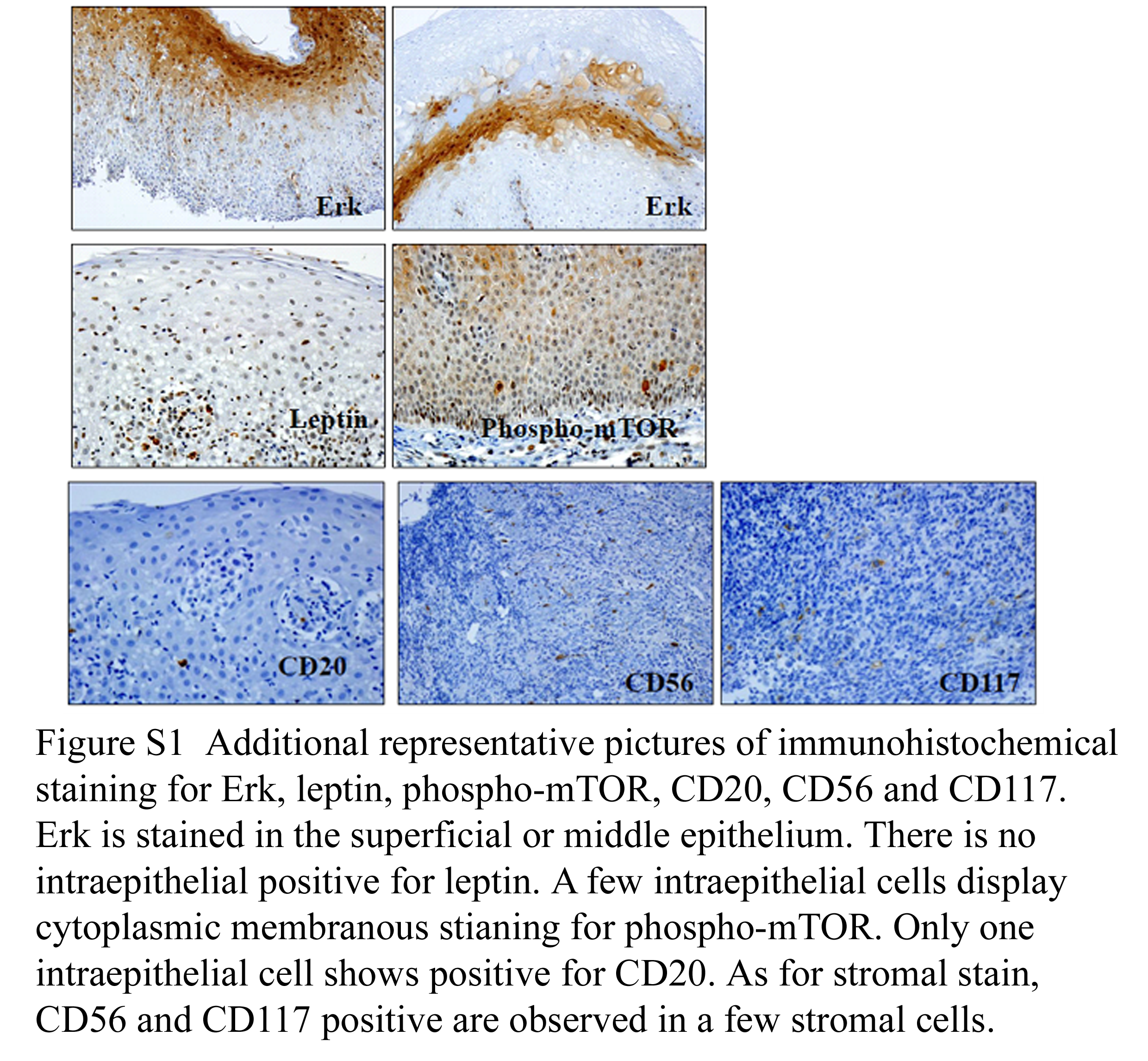

Supplement: S1 Fig — ERK is stained in the superficial or middle epithelium. There is no intraepithelial positive for leptin. A few intraepithelial cells display cytoplasmic membranous staining for phospho-mTOR. Only one intraepithelial cell shows positive for CD20. As for stromal stain, CD56 and CD117 positive are observed in a few stromal cells. (TIF) [file pone.0128110.s001.tif]
