# Supplementary material for: Histomorphological and Immunophenotypic Features of Pill-Induced Esophagitis
Source: PLoS One. 2015 Jun 5;10(6):e0128110. doi: 10.1371/journal.pone.0128110 (PMC4457729; doi:10.1371/journal.pone.0128110)
Supplement: S1 Table — (DOC) [file pone.0128110.s002.doc]

Table S1. List of treatment medicine in pill-induced esophagitis

Patient no. Treatment medicine (after discontinuing causative drugs)

| 1 | esomeprazole, almagate |
| --- | --- |
| 2 | pantoprazole, gastrexe |
| 3 | pantoprazole |
| 4 | rabeprazole, gastrex |
| 5 | lansoprazole, lamina G, gasmotin, stillen |
| 6 | sucralfate |
| 7 | no medication |
| 8 | ranitidine, sucralfate |
| 9 | famotidine, sucralfate |
| 10 | no medication |
| 11 | omeprazole, gastrex |
| 12 | famotidine, sucralfate |
| 13 | ranitidine, stillen |
| 14 | famotidine |
| 15 | lansoprazole, gastrex |
| 16 | almagate |
| 17 | pantoprazole, sucralfate |
| 18 | esomeprazole, sucralfate |
| 19 | sucralfate |
| 20 | omeprazole |
| 21 | esomeprazole, sucralfate |
| 22 | no medication |
